# Seroprevalence of viral infections in captive rhesus and cynomolgus macaques

**DOI:** 10.5194/pb-6-1-2019

**Published:** 2019-03-26

**Authors:** Artur Kaul, Uwe Schönmann, Stefan Pöhlmann

**Affiliations:** 1 Infection Biology Unit, German Primate Center – Leibniz Institute for Primate Research, 37077 Göttingen, Germany; 2 Laboratory Animal Sciences Unit, German Primate Center, 37077 Göttingen, Germany; 3 Faculty of Biology and Psychology, University of Göttingen, 37073 Göttingen, Germany

## Abstract

Macaques serve as important animal models for biomedical research. Viral infection of macaques can compromise animal health as well as the results of biomedical research, and infected animals constitute an occupational health risk. Therefore, monitoring macaque colonies for viral infection is an important task. We used a commercial chip-based assay to analyze sera of 231 macaques for the presence of antibody responses against nine animal and human viruses. We report high seroprevalence of cytomegalovirus (CMV), lymphocryptovirus (LCV), rhesus rhadinovirus (RRV) and simian foamy virus (SFV) antibodies in all age groups. In contrast, antibodies against simian retrovirus type D (SRV/D) and simian T cell leukemia virus (STLV) were detected only in 5 % and 10 % of animals, respectively, and were only found in adult or aged animals. Moreover, none of the animals had antibodies against herpes B virus (BV), in keeping with the results of in-house tests previously used for screening. Finally, an increased seroprevalence of measles virus antibodies in animals with extensive exposure to multiple humans for extended periods of time was observed. However, most of these animals were obtained from external sources, and a lack of information on the measles antibody status of the animals at the time of arrival precluded drawing reliable conclusions from the data. In sum, we show, that in the colony studied, CMV, LCV, RRV and SFV infection was ubiquitous and likely acquired early in life while SRV/D and STLV infection was rare and likely acquired during adulthood.

## 1 Introduction

Nonhuman primates (NHPs) are genetically closely related to humans and display a similar physiology. As a consequence, NHPs serve as important animal model systems for biomedical research (Gardner and Luciw, 2008). In the wild, NHPs are infected by animal viruses that may threaten NHP health and several of these viruses have zoonotic potential and also pose a threat to human health (Calvignac-Spencer et al., 2012; Cooper and Nunn, 2013; Davies and Pedersen, 2008). Moreover, the transmission of human viruses to NHPs can cause diseases in afflicted animals (Kaur et al., 2008; Messenger et al., 2014). The infection of NHPs in research facilities by animal and human viruses can compromise experiments and constitutes an occupational safety risk. Therefore, the development of diagnostics for viral infection of NHPs is an important task and is a prerequisite for the establishment of pathogen-free colonies (Yee et al., 2016).

The following animal and human viruses are of relevance regarding the monitoring of NHP colonies for viral infections: the measles virus (MV), a human virus, can be transmitted to NHPs (Willy et al., 1999; Choi et al., 1999; MacArthur et al., 1979; McChesney et al., 1989; Potkay et al., 1966; Levy and Mirkovic, 1971; Remfry, 1976) and can cause fatal disease in marmosets (*Callithrix jacchus*) (Albrecht et al., 1980; Levy and Mirkovic, 1971; Fraser et al., 1978) and macaques (Choi et al., 1999; MacArthur et al., 1979; McChesney et al., 1989; Albrecht et al., 1977; Remfry, 1976), although morbidity and mortality in macaques vary. The herpes B virus (BV, *Macacine alphaherpesvirus 1*) naturally infects macaques, which usually do not develop disease, but the transmission of the virus to other NHPs and humans can induce severe disease (Eberle and Jones-Engel, 2017, 2018). Moreover, infection by the simian immunodeficiency virus (SIV) and simian type D retroviruses (SRV/D) can cause immunodeficiency in NHPs (Schmitz and Korioth-Schmitz, 2013; Daniel et al., 1984; Marx et al., 1984; Stromberg et al., 1984) and SIV transmission from African NHPs to humans gave rise to HIV/AIDS (Sharp and Hahn, 2010). Evidence for SRV/D transmission to humans has also been obtained but has so far not been associated with disease (Lerche et al., 2001). Two other simian retroviruses – the simian T cell leukemia virus (STLV) and the simian foamy virus (SFV) – might not induce disease in NHPs and humans, but transmission to humans has been documented (Centers for Disease Control and Prevention, 1997; Betsem et al., 2011; Jones-Engel et al., 2008; Mouinga-Ondeme et al., 2012; Switzer et al., 2012; Mossoun et al., 2017; Rua and Gessain, 2015; Kazanji et al., 2015), and STLV might be the origin of human T cell leukemia virus (HTLV) (Gessain, 2011), the causative agent of T cell leukemia/lymphoma and HTLV-1 associated myelopathy/tropical spastic paraparesis. Finally, the simian cytomegalovirus (CMV, a beta-herpesvirus), the lymphocryptovirus (LCV, a gamma 1 herpesvirus and the NHP homologue of the Epstein Barr virus of humans) and the rhesus rhadinovirus (RRV, a gamma 2 herpesvirus) are frequently found in NHPs and infection is usually not associated with overt signs (Barry and William Chang, 2007; Muhe and Wang, 2015; Estep and Wong, 2013), although immunodeficient animals can develop severe disease.

Here, we report the screening of colonies of rhesus (*Macaca mulatta*) and cynomolgus macaques (*Macaca fascicularis*) housed at the German Primate Center (Deutsches Primatenzentrum, DPZ) for antibody responses against retro- (SRV/D, SIV, STLV, SFV), herpes (BV, LCV, CMV, RRV) and paramyxoviruses (MVs).

## 2 Materials and methods

### 2.1 Sample collection and storage

Blood serum samples were collected during the annual health monitoring in 2017. Subsequently, the samples were stored at -20 ${}^{\circ }$C until testing.

### 2.2 Detection of antibodies against viruses

The detection of the specific serum immunoglobulin G (IgG) to BV, SIV, SRV/D, STLV, SFV, RRV, LCV, CMV and MV was performed in a multiplex format using the Simian Panel E Kit (Intuitive Biosciences, USA) according to the manufacturer's instructions and using only materials provided by the manufacturer. In brief, blood serum was thawed and vortexed. Afterwards, serum was diluted 1:400 using the diluent buffer (CSA buffer, Simian Panel E Kit), and 100 µL of diluted serum was added per well of the array. After an incubation of 60 min at room temperature, the arrays were washed three times and then incubated with a gold conjugated secondary antibody. Subsequently, the arrays were washed three times and reagents for gold-catalyzed silver deposition were added. After 3 min of incubation, signals were detected using the image capture and analysis system AQ 1000 and quantified using Athena Quant software (Intuitive Biosciences, USA). As reviewed recently (Yee et al., 2016), the assay has been validated by the manufacturer using previously well-characterized negative and positive serum samples.

### 2.3 Ethics statement

The animals were housed and treated under standard conditions, which are in accordance with the German Animal Welfare Act and the European Union guidelines on the use of nonhuman primates for biomedical research and the Weatherall report. According to §11 of the German Animal Welfare Act, the DPZ is permitted to breed and house nonhuman primates under license number 392001/7 issued by the local veterinary authorities. Samples were collected during the annual health monitoring, which is part of colony management and does not require approval by regulatory agencies or an ethic committee.

### 2.4 Statistical analysis

Statistical analysis was performed using the Graphpad Prism7 software. All 2×2 contingencies within Table 1 were analyzed by means of Fisher's exact test. Trend developments of the age classes (last row of Table 1) were calculated by means of the chi-square test for trend. Both tests were two-sided with the level of statistical significance of 0.05.

**Table 1:** Seroprevalence of simian viruses in macaques; DPZ, 2017.

		Seroprevalence, % (no. positive)								
Category	No. (%)	BV	SIV	STLV	SRV	Measles	CMV	LCV	RRV	SFV
Species										
*M. mulatta*	199 (86.1)	0.0 (0)	0.0 (0)	0.0 (0)	1.0 (2)	7.5 (15)	99.5 (198)	89.4 (178)	84.9 (169)	100 (199)
*M. fascicularis*	32 (13.9)	0.0 (0)	0.0 (0)	6.3 (2)	12.5 (4)	34.4 (11)	90.6 (29)	90.6 (29)	81.3 (26)	62.5 (20)
	P value	–	–	0.0187	0.0038	0.0001	0.0089	>0.9999	0.6015	<0.0001
Sex${}^{2}$										
Male	74 (32.0)	0.0 (0)	0.0 (0)	0.0 (0)	0.0 (0)	13.5 (10)	100 (74)	90.5 (67)	77.0 (57)	96.0 (71)
Female	156 (67.5)	0.0 (0)	0.0 (0)	1.3 (2)	3.8 (6)	10.3 (16)	97.4 (152)	77.6 (121)	87.8 (137)	94.2 (147)
	P value	–	–	>0.9999	0.1806	0.5064	0.3082	0.0177	0.0508	0.7559
Housing conditions${}^{3}$										
Inside area	45 (19.5)	0.0 (0)	0.0 (0)	4.4 (2)	6.7 (3)	40.0 (18)	100 (45)	93.3 (42)	88.4 (38)	95.6 (43)
Outside areas	186 (80.5)	0.0 (0)	0.0 (0)	0.0 (0)	1.6 (3)	4.3 (8)	97.9 (182)	88.7 (165)	84.4 (157)	94.6 (176)
	P value	–	–	0.0373	0.0899	<0.0001	>0.9999	0.5852	>0.9999	>0.9999
Age class${}^{1}$										
Infant	4 (1.7)	0.0 (0)	0.0 (0)	0.0 (0)	0.0 (0)	0.0 (0)	75.0 (3)	100 (4)	100 (4)	50.0 (2)
Juvenile	37 (16.0)	0.0 (0)	0.0 (0)	0.0 (0)	0.0 (0)	0.0 (0)	94.6 (35)	91.9 (34)	73.0 (27)	78.4 (29)
Adolescent	47 (20.4)	0.0 (0)	0.0 (0)	0.0 (0)	0.0 (0)	0.0 (0)	97.9 (46)	83.0 (39)	78.7 (37)	100 (47)
Adult	104 (45.0)	0.0 (0)	0.0 (0)	0.0 (0)	1.9 (2)	10.6 (11)	100 (104)	90.4 (94)	92.3 (96)	98.1 (102)
Aged	39 (16.9)	0.0 (0)	0.0 (0)	5.1 (2)	10.3 (4)	38.5 (15)	100 (39)	92.3 (36)	79.5 (31)	100 (39)
	P value	–	–	0.0460	0.0078	<0.0001	0.0013	0.7905	0.1831	<0.0001
Total	231	0.0 (0)	0.0 (0)	0.9 (2)	2.6 (6)	11.3 (26)	98.3 (227)	89.6 (207)	84.4 (195)	94.8 (219)

${}^{1}$ Age class determinations: infant, 0.5–1.5 years of age; juvenile, 1.5–3.5 years; adolescent, 3.5–5 years; adult, 5–15 years; aged, ≥15 years. ${}^{2}$ Sex is missing for one animal. ${}^{3}$ Group housing. Statistical analysis was performed using the GraphPad Prism7 software. The chi-square test for trend was applied for the groups of the age classes and Fisher's exact test for all remaining groups. Both tests were two-sided with the level of statistical significance of 0.05. Corresponding p values are depicted below the tested groups.

## 3 Results

We analyzed the antibody status of 231 macaques kept at the German Primate Center for biomedical research and for allocation to other scientific institutions. The majority of the animals (86.1 %) were rhesus macaques, while the remaining animals (13.9 %) were cynomolgus macaques (Table 1). Roughly two-thirds of the animals were females and approximately 60 % of the animals were born at the German Primate Center while the rest was obtained from international providers. Breeding colonies had access to outside enclosures (outside area), while animals used or to be used in experimental research had limited or no exposure to outside conditions (inside area).

A commercial chip-based screening system was used to detect antibodies against SRV/D, SIV, STLV, SFV, BV, CMV, RRV, LCV and MV. The animals had previously been tested negative for BV antibodies using in-house assay systems (Pöhlmann et al., 2017) and were also found to be negative employing the commercial detection system (Table 1). Similarly, all animals were negative for SIV antibodies. Two animals were positive for STLV antibodies, and six animals harbored antibodies against SRV/D, all of them adult or aged females. In contrast, between 90 % to 100 % of the adult animals were seropositive for the herpesviruses CMV, LCV and RRV as well as for the retrovirus SFV, and seroprevalence of CMV and SFV significantly increased with age (Table 1). However, it cannot be excluded that seropositivity of infants was due to the presence of maternal antibodies. Finally, roughly 11 % of the animals had antibodies against MV and seropositivity of animals was significantly higher in housing and experimental situations entailing more extensive and extended contact with humans (inside area) than in the breeding groups (outside area).

## 4 Discussion

Information on the infection status of NHP colonies kept for biomedical research is vital for the maintenance of animal health and for the adequate interpretation of experimental results. Here, we used a chip-based platform to analyze macaque colonies for the presence of blood serum antibodies against eight NHP viruses (SRV/D, SIV, STLV, SFV, BV, CMV, LCV, RRV) and a human virus (MV) that constitutes a threat to NHPs and human health. Our results indicate that infection with the herpesviruses CMV, RRV and LCV as well as the retrovirus SFV is frequent in the animal population studied and suggest that previous efforts, based on testing with in-house assays for BV-specific antibodies (Pöhlmann et al., 2017), to establish a BV-free colony were successful. Finally, our results suggest a potential transmission of MV to NHPs enrolled in research programs that involve frequent animal–human contact, although the unknown MV vaccination status of the NHPs in question precludes solid conclusions.

Our results indicate that infection by the herpesviruses CMV, LCV and RRV and by the retrovirus SFV is frequent and often acquired early in life in the macaque population studied. These results are generally in keeping with published data (Chu et al., 1971; Ishida and Varavudhi, 1992; Ryan and Rose, 2013; Vogel et al., 1994; Calattini et al., 2006; Jones-Engel et al., 2007, 2008; Muniz et al., 2015) although the macaque colony in Gibraltar was found to be CMV negative, for reasons not fully understood (Engel et al., 2008). Transmission is thought to occur via the oral route and/or through bites and scratches. Finally, SFV can be transmitted to humans (Rua and Gessain, 2015; Stenbak et al., 2014; Muniz et al., 2017; Jones-Engel et al., 2008), indicating that animals and animal material should be handled with appropriate protective measures, although at present no diseases haven been linked to zoonotic transmission events.

SIV, STLV, BV and potentially also SRV/D infection may compromise animal and/or human health. In particular, the transmission of BV from macaques to humans can cause encephalitis that may take a fatal course in 70 %–80 % of afflicted patients in the absence of treatment (Eberle and Jones-Engel, 2018). Therefore, the antibody status of animals is determined before purchase and use in experimentation (SIV, STLV, BV and SRV/D) and/or is continuously monitored (BV). However, the detection of BV-specific antibodies is challenging since antibody responses may develop only months after infection and antibody titers in infected animals maybe very low or undetectable at certain times after infection (Olson et al., 1991; Ward and Hilliard, 2002; Wolfensohn and Gopal, 2001). We had previously used an in-house Enzyme Linked Immunosorbent Assay (ELISA) based on lysates from cells infected with herpesvirus papio 2 or a modified anti-HSV ELISA kit (Enzygnost^®^ Anti-HSV/IgG, Siemens) (Pöhlmann et al., 2017) to detect antibodies against BV in the colony studied here with negative results. Therefore, the finding that none of the macaques had BV antibodies as determined in the chip-based approach suggests that the in-house ELISAs might be highly sensitive.

MV infection of macaques recapitulates important aspects of MV pathogenesis and immune responses in humans, and the virus can be transmitted between animals (de Swart, 2009; de Vries et al., 2012; Choi et al., 1999; MacArthur et al., 1979; McChesney et al., 1989; Potkay et al., 1966; Remfry, 1976; Willy et al., 1999). We observed a significantly higher prevalence of MV antibodies in macaques in housing and experimental situations entailing more extensive and extended contact with humans (inside area, Table 1) than in the breeding groups (outside area, Table 1). In total, 17 out of the 18 MV-antibody-positive animals housed in inside areas came from external sources and the MV antibody status of these animals at time of arrival at DPZ is not known (some of these animals arrived at DPZ in 2003; others had been at DPZ since 2012 and 2016, respectively). Moreover, for none of the antibody-positive animals were measles signs detected. Thus, it is possible that the animals were vaccinated or exposed to MV before arrival at DPZ. However, to our knowledge, the external sources do not routinely conduct measles vaccination and 11 out of 12 macaques obtained from one of these sources but not exposed to extensive animal–human contact at DPZ were seronegative. Alternatively, the animals might have acquired an MV infection at DPZ, but several points argue against this possibility: first, the measles vaccination status of all DPZ personnel working with NHPs is controlled, including the analysis of antibody titers. Second, personal protective equipment is used, including gloves, facial protection, safety goggles, lab shoes and a lab coat. Third, no case of acute MV infection among DPZ personnel with access to NHPs has been recorded. Therefore, the reason for the increased MV antibody seropositivity in animals with extensive contact to humans could not be established by the present study, and MV transmission from humans to animals despite safety measures is only one of several potential explanations.

## Data Availability

All relevant data are presented in the paper. For additional information, please contact the corresponding authors.
